# Nicotinamide mononucleotide improves energy activity and survival rate in an *in vitro* model of Parkinson’s disease

**DOI:** 10.3892/etm.2014.1842

**Published:** 2014-07-14

**Authors:** LEI LU, LE TANG, WENSHI WEI, YUNYI HONG, HEYU CHEN, WEIHAI YING, SHENGDI CHEN

**Affiliations:** 1 Department of Neurosurgery, Huashan Hospital, Fudan University, Shanghai 200040, P.R. China; 2 School of Biomedical Engineering and Med-X Research Institute, Shanghai Jiao Tong University, Shanghai 200030, P.R. China; 3 Department of Neurology, Huadong Hospital, Fudan University, Shanghai 200040, P.R. China; 4 Department of Neurology and Institute of Neurology, Ruijin Hospital, School of Medicine, Shanghai Jiao Tong University, Shanghai 200025, P.R. China

**Keywords:** nicotinamide mononucleotide, Parkinson’s disease, energy activity, cell survival

## Abstract

Nicotinamide adenine dinucleotide (NAD^+^) repletion has been shown to provide marked neuroprotection from genotoxic agent-induced neuronal and astrocyte cell death. One of the key precursors of NAD^+^ is nicotinamide mononucleotide (NMN). Therefore, it was hypothesized that NMN may attenuate apoptosis and improve energy metabolism in Parkinson’s disease (PD)-like behavioral and neuropathological changes, and produce significant beneficial effects. In this study, a cellular model of PD, using rotenone-treated PC12 cells, was established to test the hypothesis that NMN may decrease PD-like pathological changes. Experiments were carried out to investigate cell survival, including an intracellular lactate dehydrogenase (LDH) assay. Apoptotic and necrotic cell death, NAD^+^ levels and ATP levels were also evaluated. It was observed that NMN was able to significantly attenuate the rotenone-induced reduction in the survival rate of PC12 cells, as assessed by MTT and LDH assays. NMN treatment also significantly reduced the rotenone-induced apoptosis of the cells, as assessed by flow cytometry-based Annexin V/7-aminoactinomycin D staining. Furthermore, NMN restored intracellular levels of NAD^+^ and ATP in the rotenone-treated cells, thus demonstrating the capacity of NMN to ameliorate mitochondrial inhibitor-induced impairments of energy metabolism. The present study indicates that NMN produces significant beneficial effects by attenuating apoptosis and improving energy metabolism in a cellular model of PD. These results suggest that NMN may become a promising therapeutic drug for PD.

## Introduction

A number of studies have demonstrated that nicotinamide adenine dinucleotide (NAD^+^) plays an important role in energy metabolism and mitochondrial function, as well as gene expression, calcium homeostasis, aging and cell death (1–3). Multiple studies have also revealed that NAD^+^ is a cytoprotective agent for primary cultures of astrocytes, neurons and myocytes; NAD^+^ treatment has been shown to decrease the necrotic cell death in these types of cells induced by oxidative stress (4), DNA-alkylating agents (5) or oxygen-glucose deprivation (6). A previous study demonstrated that NAD^+^ administration significantly reduced ischemic brain injury in rats (7). One of the key precursors of NAD^+^ is nicotinamide mononucleotide (NMN), which is converted to NAD^+^ by nicotinamide mononucleotide adenylyltransferase (2).

Parkinson’s disease (PD) is one of most common types of movement disorder. A major pathological change of the disease is the loss of dopaminergic neurons in the substantia nigra pars compacta (8). Although it has been suggested that oxidative stress, mitochondrial dysfunction, inflammation or apoptosis of the dopaminergic neurons in the substantia nigra may play a key role in the pathogenesis of PD (9), the precise mechanisms underlying the pathogenesis of PD are not well understood (10). There has been little effective therapy for treatment of this debilitating disorder (11). Dysfunction of the mitochondria may be involved in the pathogenesis of PD and may become a promising therapeutic target for the disease (12).

*In vitro* and *in vivo* studies have revealed that rotenone, a mitochondrial complex I inhibitor, induces PD-like behavioral and neuropathological changes, including the induction of apoptosis and acceleration of α-synuclein formation, in PD models (13,14). Since NAD^+^ treatment is able to attenuate the genotoxic agent-induced mitochondrial alterations in neurons and astrocytes (4), it was hypothesized that NMN treatment may attenuate rotenone-induced cytotoxicity. In the current study, a cellular model of PD, using rotenone-treated PC12 cells, was established to investigate whether NMN is a protective agent against rotenone-induced cytotoxicity.

## Materials and methods

### Cell culture

PC12 cells were purchased from the Cell Resource Center of the Shanghai Institute of Biological Sciences of the Chinese Academy of Sciences (Shanghai, China). The cells were plated at an initial density of 5×10^4^ cells/well onto 24-well culture plates to test cell viability, and at a density of 1×10^6^ cells/well onto 6-well plates in order to prepare samples for western blot analysis. The culture media used was Dulbecco’s modified Eagle’s medium containing 4,500 mg/l D-glucose, 584 mg/l L-glutamine and 110 mg/l sodium pyruvate (Thermo Scientific, Tewksbury, MA, USA), and also containing 1% penicillin and streptomycin (Invitrogen Life Technologies, Carlsbad, CA, USA) and 10% fetal bovine serum (PAA Laboratories GmbH, Linz, Austria). The cells were given 0.5 μM rotenone (Sigma, St. Louis, MO, USA) with or without co-treatment with different concentrations of NMN (N3501, Sigma Aldrich, USA). The cells were kept for 24 h in an incubator with 5% CO_2_ at 37°C. In total, the following four concentrations were tested: 0.1mM, 1.0 mM, 5mM and 10mM, however, the effects of last three appeared to be similar. Thus, the minimum (0.1 mM) and maximum (1.0 mM) concentrations were selected in order to indicate the effect of improving the energy activity and survival rate of rotenone-treated PC12 cells/

### Determination of cell survival

Cell survival was measured by a quantitative colorimetric assay with 3-(4,5-dimethylthiazol-2-yl)-2,5-diphenyltetrazolium bromide (MTT; Sigma). Following drug treatment, PC12 cells were incubated for 4 h with 5 mg/ml MTT. Subsequently the cell cultures were lysed in dimethyl sulfoxide (DMSO; Sigma) for 15 min. The optical absorption at 570 nm was determined using a plate reader (Synergy2, Biotek, Winooski, VT, USA).

### Intracellular lactate dehydrogenase (LDH) assay

Using a previously described method (15), cell survival was quantified by the measurement of LDH activity in cell lysates. Cells were lysed for 20 min in lysing buffer containing 0.04% Triton X-100, 2 mM 4-(2-hydroxyethyl)-1-piperazineethanesulfonic acid (HEPES), 0.2 mM dithiothreitol, 0.01% bovine serum albumin and 0.1% phenol red. A total of 50 μl cell lysates at pH 7.5 were mixed with 150 μl 500 mM potassium phosphate buffer (pH 7.5) containing 1.5 mM NADH (Sigma) and 7.5 mM sodium pyruvate (Sigma). The change in the absorbance at 340 nm (A340 nm) was monitored over 90 sec. Percentage cell survival was calculated by standardizing the LDH activity of the sample cell lysates to the LDH activity of the lysates of the control (wash only) cell cultures.

### Extracellular LDH assay

LDH is a cytosolic enzyme that is released into the cell media upon cell lysis. Extracellular LDH activity is highly correlated with a major index of cell necrosis: the level of propidium iodide-positive cells (16). Therefore, extracellular LDH activity may be used for assessing the number of cells undergoing cell death (17). In the present study, the extracellular LDH activity of PC12 cells was assessed using a previously described method (16). Briefly, 100 μl extracellular media from the samples was mixed with 150 μl potassium phosphate buffer (500 mM, pH 7.5) containing 1.5 mM NADH and 7.5 mM sodium pyruvate. Changes in the A340 nm of the samples was monitored over 90 sec using a plate reader.

### Determination of apoptotic and necrotic cell death by flow cytometry-based Annexin V/7-aminoactinomycin D (7-AAD) staining

Following washing twice with phosphate-buffered saline (PBS), the cells were suspended in cold 1X binding buffer at a concentration of 1×10^6^ cells/ml. A total of 100 μl cell suspension was mixed with 10 μl Annexin V R-phycoerythrin (PE) conjugate (SouthernBiotech, Birmingham, AL, USA). Following incubation on ice for 15 min, 200 μl cold 1X binding buffer was added to each tube, followed by the addition of 10 μl 7-AAD (SouthernBiotech). The number of the cells in early- and late-stage apoptosis, and necrosis, was assessed by a flow cytometer (BD FACSAria II; BD Biosciences, Franklin Lanes, NJ, USA).

### Hoechst 33258 staining

The nuclear size of cells was assessed by Hoechst staining (18). Following treatment, cells were fixed in 4% formaldehyde for 15 min at room temperature. After washing with PBS, the cells were stained with 20 μg/ml Hoechst 33258 (Sigma) in PBS for 20 min. Images of the stained nuclei were captured under a fluorescence microscope (Leica DMI3000B, Leica, Mannheim, Germany).

### Western blot analysis of poly (ADP-ribose) polymerase 1 (PARP-1)

Using a previously described method (19), PC12 cells were lysed in radioimmunoprecipitation assay (RIPA) cell lysis buffer [50 mM Tris-HCl, pH 8.0, 150 mM NaCl, 1% NP-40, 0.5% sodium deoxycholate and 0.1% sodium dodecyl sulfate (SDS); Sigma] supplemented with 100 μM protease inhibitor cocktail (Roche Diagnostics, Mannheim, Germany). Membrane fractions were separated by centrifugation at 16,000 × g for 20 min at 4°C. Protein concentrations were determined using a bicinchoninic acid (BCA) protein assay kit (Thermo Scientific, Rockford, IL, USA). The samples were normalized to 30-μg of total protein extract and were fractionated by 10% SDS-polyacrylamide gel electrophoresis and transferred onto polyvinylidene difluoride membranes using a standard technique. The membranes were blocked with 5% skimmed milk at 37°C for 1 h and incubated with rabbit PARP-1 (p116/p25) antibodies (1:1,000; Epitomics, Burlingame, CA, USA) at 4°C overnight. Horseradish peroxidase (HRP)-linked anti-rabbit immunoglobulin G (IgG; Epitomics) and enhanced chemiluminescence (ECL) substrate (Thermo Scientific) were added and the bands were visualized. The equal loading of samples was confirmed by stripping the membranes and reprobing them with goat polyclonal anti-actin IgG (1:400, Santa Cruz Biotechnology, Inc., Santa Cruz, CA, USA) and HRP-conjugated rabbit anti-goat IgG (Epitomics). Levels of immunoreactive proteins were determined by densitometric scanning using a ChemiDoc XRS system (Bio-Rad, Hercules, CA, USA).

### Determination of the levels of ATP

ATP levels were quantified using an ATP Bioluminescence assay kit HS II (Roche Diagnostics) according to the manufacturers’ instructions. Cells were lysed with cell lysis reagent (Roche Diagnostics) and 50 μl lysates were mixed with 150 μl luciferase assay reagent (Roche Diagnostics). The luminescence was detected using a plate reader (Biotek Synergy 2; BioTek Instrument, Inc., Winooski, VT, USA). The protein concentrations of the samples were determined by a BCA assay. The ATP concentrations of the samples were calculated using an ATP standard and normalized against the amount of protein in the samples.

### NAD^+^ assay

Following drug treatment, the cells were carefully washed with PBS. The levels of NAD^+^ were measured using a plate reader according to a previously described method (4). Cells were extracted in 0.25 ml 0.5 N HClO_4_, scraped, neutralized with 3 M KOH and 100 mM sodium phosphate buffer (pH 7.0). The levels of NAD^+^ were assessed based on the reduction of MTT to formazan by NADH, which was generated by enzymatic cycling with alcohol dehydrogenase. The rate of optical density (OD) increase at 560 nm was determined by examining the samples immediately and 20 min after the addition of the sample extracts.

### Statistical analyses

All data are presented as mean + standard error. Data were assessed by one-way analysis of variance (ANOVA) followed by the Student-Newman-Keuls post hoc test. P<0.05 was considered to indicate a statistically significant difference.

## Results

### Treatment with NMN attenuates rotenone-induced injury of PC12 cells

**Figure 1:**
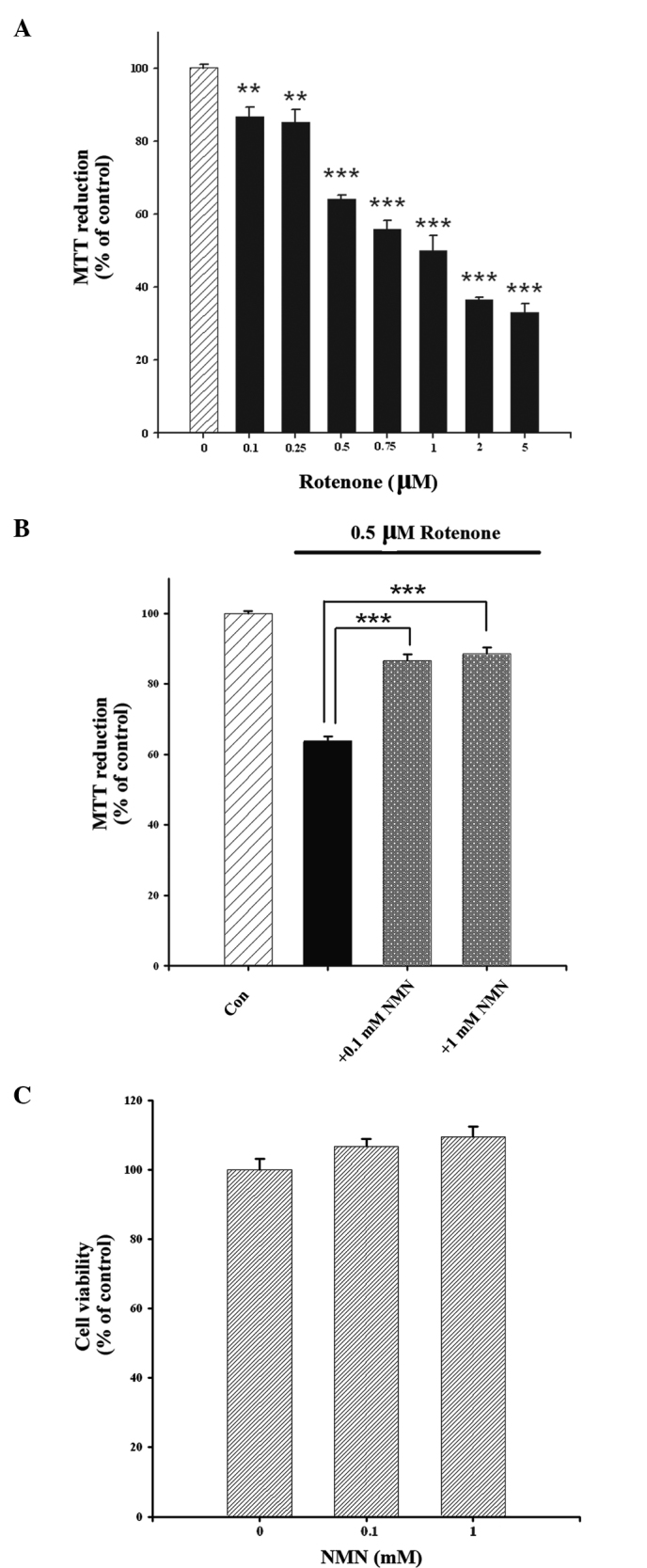
Nicotinamide mononucleotide (NMN) treatment significantly attenuates the rotenone-induced reduction in the survival rate of PC12 cells. (A) Rotenone concentration-dependently decreased the survival rate of PC12 cells, as assessed by an 3-(4,5-dimethylthiazol-2-yl)-2,5-diphenyltetrazolium bromide (MTT) assay. Following treatment with various concentrations of rotenone for 24 h, the PC12 cell survival rate was determined by MTT assay. (B) NMN treatment significantly attenuated the rotenone-induced reduction in the survival rate of PC12 cells, as assessed by MTT assay; the PC12 cells were co-treated with rotenone and NMN for 24 h. (C) PC12 cells treated with NMN alone. Subsequent cell survival rate was determined by MTT assay. N=12, N, the number of wells for each concentration. Data were collected from ≥3 independent experiments. ^**^P<0.01; ^***^P<0.001 as indicated, or vs. control (Con).

The present study determined the effect of NMN treatment on rotenone-induced changes in the survival rate of PC12 cells using MTT, intracellular LDH and extracellular LDH assays. Treatment with rotenone for 24 h decreased the survival rate of PC12 cells in a concentration-dependent manner, as assessed by MTT assay (Fig. 1A). Since 0.5 μM rotenone induced an ~60% decrease in MTT reduction, this concentration of rotenone was used in subsequent experiments. Co-treatment of the cells with NMN (0.1 or 1 mM) and rotenone led to a significantly higher survival rate of the PC12 cells when compared with the survival rate of the PC12 cells treated with rotenone alone (Fig. 1B). The effect of treatment with NMN alone is shown in Fig. 1C.

**Figure 2:**
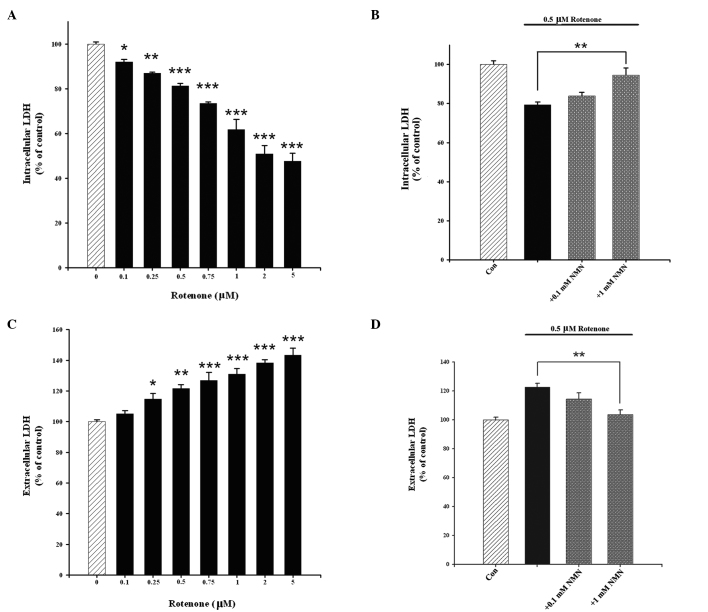
(A) Rotenone concentration-dependently decreased the survival rate of PC12 cells, as assessed by intracellular lactate dehydrogenase (LDH) assay. PC12 cells were treated with various concentrations of rotenone for 24 h. Subsequently, cell survival was determined by intracellular LDH assay. (B) Nicotinamide mononucleotide (NMN) treatment significantly attenuated the rotenone-induced reduction in the survival rate of PC12 cells, as assessed by intracellular LDH assay. (C) Rotenone concentration-dependently induced PC12 cell death, as assessed by extracellular LDH assay. (D) NMN treatment significantly attenuated the rotenone-induced PC12 cell death, as assessed by extracellular LDH assay.^*^P<0.05; ^**^P<0.01; ^***^P<0.001 as indicated or vs. 0 mM rotenone. Con, control.

An intracellular LDH assay was also conducted to determine the effects of NMN and rotenone on cell survival. Treatment of the cells with rotenone caused a concentration-dependent reduction in intracellular LDH activity, which is an index of cell survival (Fig. 2A). This effect was significantly attenuated by NMN co-treatment (Fig. 2B). Cell death was assessed by extracellular LDH assay. It was observed that rotenone concentration-dependently induced the death of the PC12 cells (Fig. 2C), which was significantly attenuated by NMN co-treatment (Fig. 2D).

### Treatment with NMN decreases rotenone-induced apoptosis of PC12 cells

**Figure 3:**
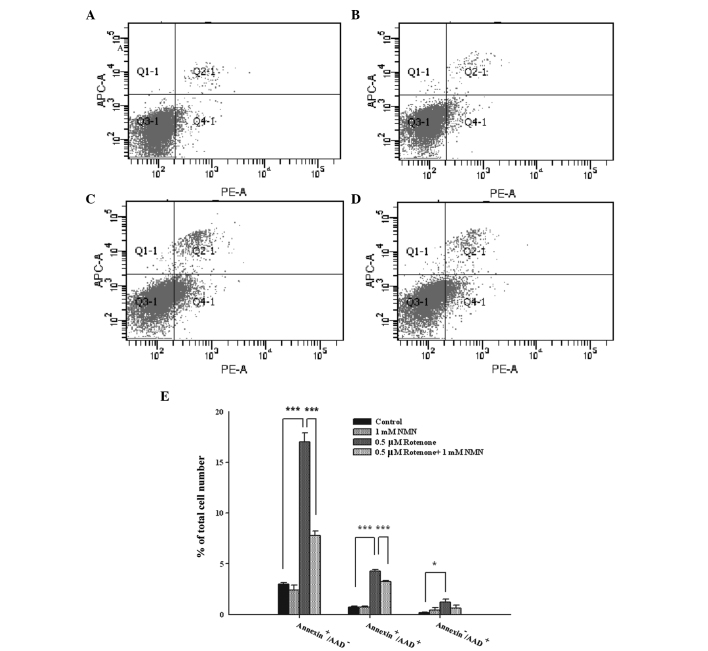
Nicotinamide mononucleotide (NMN) treatment attenuates the rotenone-induced apoptosis of PC12 cells. A flow cytometry-based Annexin V/7-aminoactinomycin D (7-AAD) staining assay was used to determine early- and late-stage apoptosis and necrosis. Treatment of the cells with (A) 1 mM NMN did not significantly affect the level of cell death when compared with (B) the control cells. (C) Treatment of cells with 0.5 mM rotenone led to an increase in the number of Annexin V+/7-AAD- and Annexin V+/7-AAD- cells, which was significantly decreased by (D) the co-treatment of the cells with NMN. (E) Quantification of the FACS results revealed that NMN treatment can significantly decrease apoptosis and necrosis of the rotenone-treated cells among the total cell number. ^*^P<0.05; ^***^P<0.001; APC, allophycocyanin; PE, phycoerythrin.

Flow cytometry-based Annexin V/7-AAD staining was conducted to determine the early and late apoptosis and necrosis of PC12 cells treated with rotenone and NMN. Phosphatidylserine (PS) is expressed on the outer leaflet of the plasma membranes of early-stage apoptotic cells, which may be stained by labeled Annexin V. Late-stage apoptotic and necrotic cells lose the integrity of their plasma membranes, which become permeable to the fluorescent dye 7-AAD (20). Thus, Annexin V^−^/7-AAD^−^, Annexin V^+^/7-AAD^−^, Annexin V^+^/7-AAD^+^ and Annexin V^−^/7-AAD^+^ cells are defined as normal, early-stage apoptotic, late-stage apoptotic and necrotic cells, respectively. Treatment of the cells with 1 mM NMN (Fig. 3A) did not significantly affect the level of cell death compared with that of the control cells (Fig. 3B). Treatment of the cells with 0.5 μM rotenone led to a marked increase in the number of Annexin V^+^/7-AAD^−^ cells, as well as increases in the numbers of Annexin V^+^/7-AAD^+^ and Annexin V^−^/7-AAD^+^ cells, compared with the control group (Fig. 3C). Co-treatment of the cells with rotenone and NMN led to significant reductions in the numbers of Annexin V^+^/7-AAD^−^ and Annexin V^+^/7-AAD^+^ cells when compared with the rotenone treatment alone (Fig. 3D). Quantification of these results indicates that NMN significantly decreases the numbers of early- and late-stage apoptotic cells (Fig. 3E).

**Figure 4:**
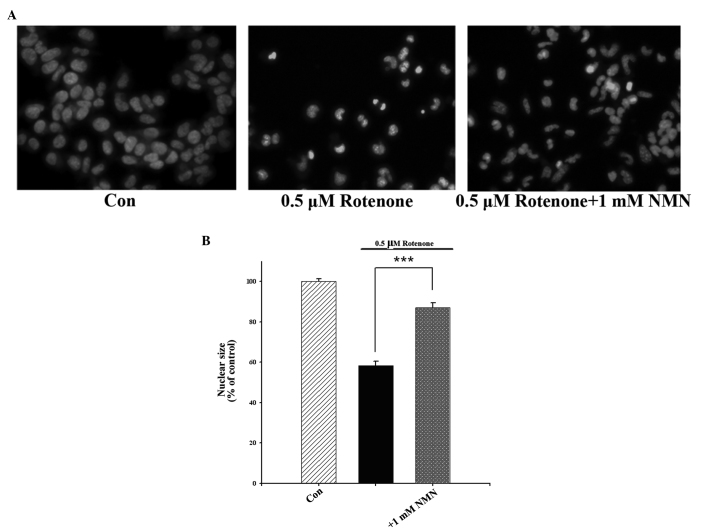
Nicotinamide mononucleotide (NMN) treatment attenuates rotenone-induced nuclear condensation, as assessed by Hoechst 33258 staining. PC12 cells were treated with 0.5 μM rotenone, with or without co-treatment with NMN for 24 h. (A) The nuclear condensation of the cells was assessed by Hoechst 33258 staining. The images presented are representative of the those captured in three independent experiments. Magnification, ×200. (B) Quantification of the nuclear size of the cells revealed that NMN treatment significantly attenuated rotenone-induced nuclear condensation. ^***^P<0.001.

**Figure 5:**
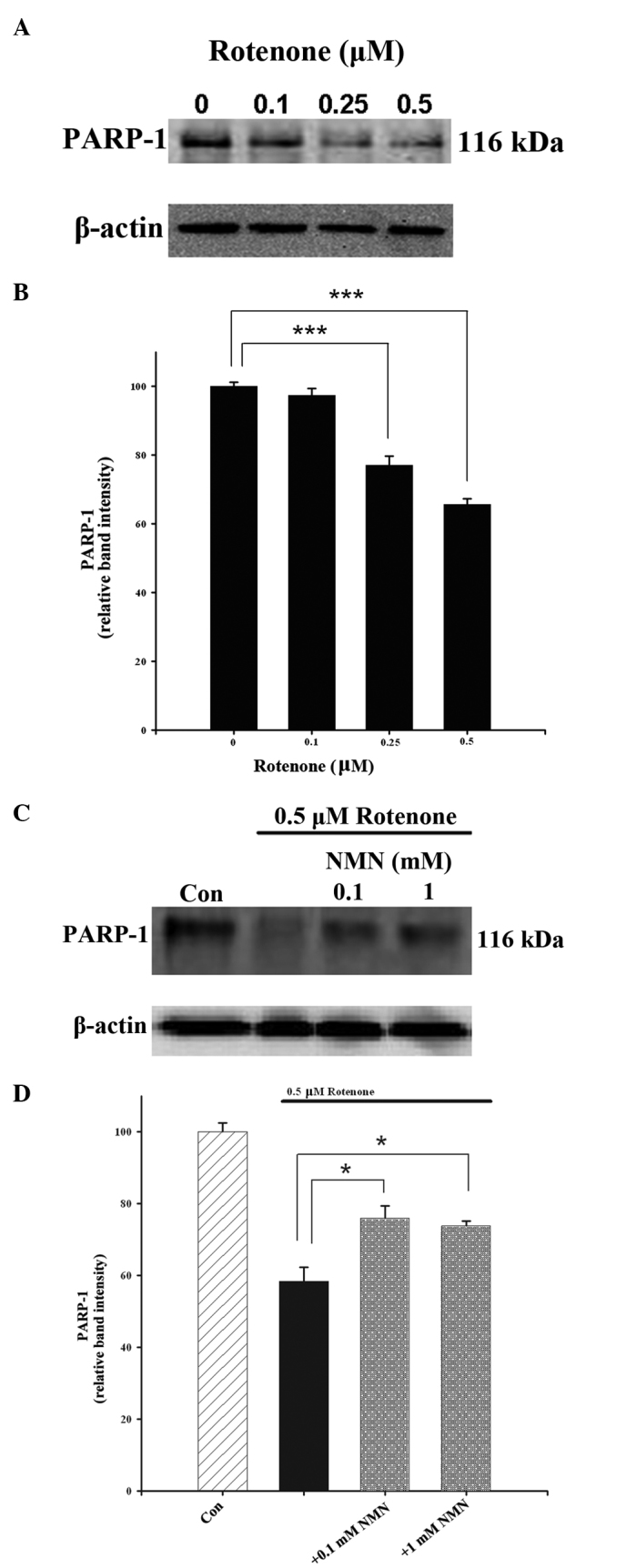
Nicotinamide mononucleotide (NMN) treatment attenuates the rotenone-induced reduction of the levels of poly (ADP-ribose) polymerase 1 (PARP-1) in PC12 cells, as determined by western blot analysis using anti-PARP-1 antibodies. PC12 cells were treated with 0.5 μM rotenone, with or without co-treatment with NMN for 24 h. (A and B) 0.5 μM rotenone caused a reduction in the levels of PARP-1, as assessed by western blot analysis. (C and D) NMN treatment significantly attenuated the rotenone-induced reduction of the levels of PARP-1. N=9, N, the times of repeats of the experiments. Data were collected from three independent experiments.^*^P<0.05; Con, control.

Nuclear condensation is an indication of cellular apoptosis and may be observed through Hoechst 33258 staining. Furthermore, PARP-1 is cleaved by active caspase-3 during caspase-3-dependent apoptotic processes. To further determine whether NMN is able to decrease rotenone-induced apoptosis, the levels of nuclear condensation and PARP-1 cleavage in rotenone-treated PC12 cells were assessed. It was observed that rotenone concentration-dependently induced nuclear condensation, and this effect was significantly attenuated by NMN co-treatment (Fig. 4). Western blot analysis also revealed that rotenone concentration-dependently reduced the levels of PARP-1 (Fig. 5A and B). The rotenone-induced reductions in the levels of PARP-1 were significantly attenuated by NMN co-treatment (Fig. 5C and D).

### Treatment with NMN restores the intracellular levels of NAD^+^ in rotenone-treated PC12 cells

**Figure 6:**
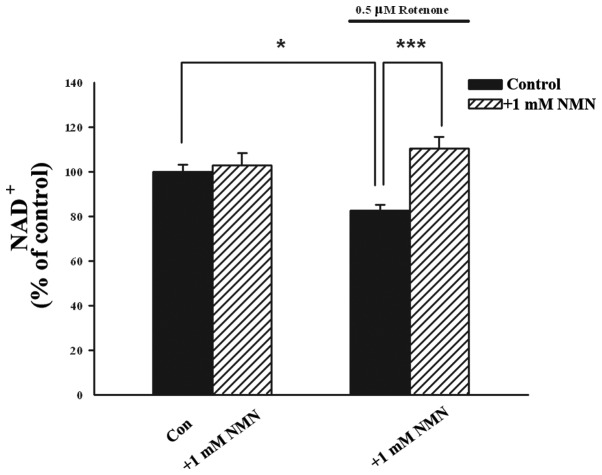
Nicotinamide mononucleotide (NMN) treatment attenuated the rotenone-induced reduction in the intracellular levels of nicotinamide adenine dinucleotide (NAD^+^). PC12 cells were treated with 0.5 mM rotenone, with or without co-treatment with 1 mM NMN for 24 h. Subsequently, the intracellular levels of NAD^+^ in the cells were determined.^*^P<0.05; ^***^P< 0.001; Con, control.

Since NMN is a precursor of NAD^+^, the present study aimed to determine whether NMN treatment enhances the intracellular levels of NAD^+^ in PC12 cells. It was observed that treatment of PC12 cells with 0.5 mM rotenone led to a significant reduction in the intracellular levels of NAD^+^, which was prevented by co-treatment with 1 mM NMN for 24 h (Fig. 6).

### Treatment with NMN attenuates the rotenone-induced reduction in intracellular levels of ATP in PC12 cells

**Figure 7:**
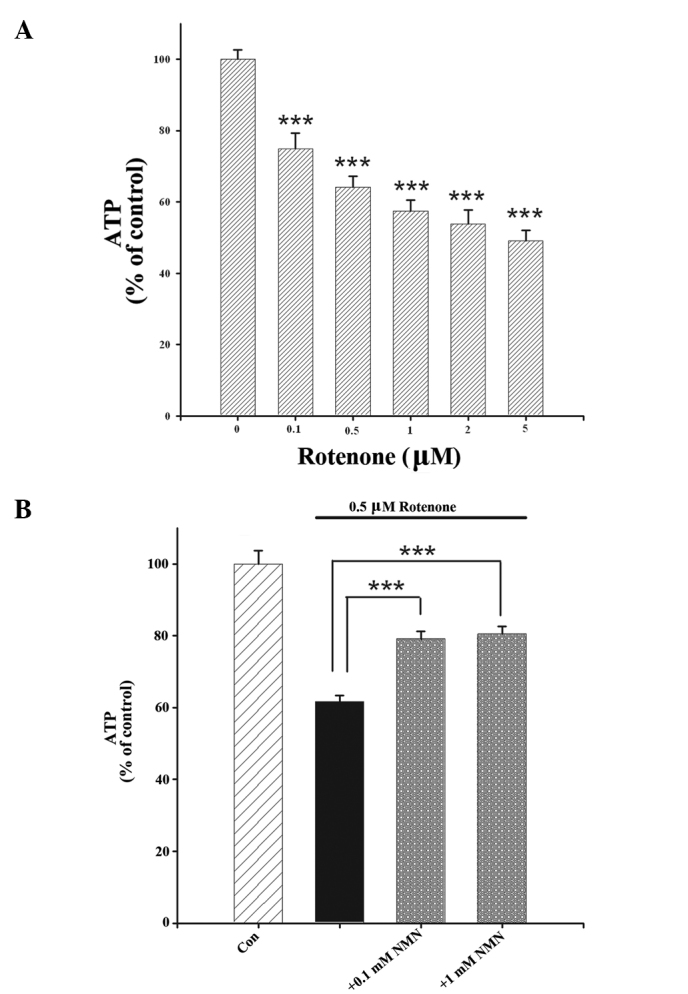
Nicotinamide mononucleotide (NMN) treatment attenuates the rotenone-induced reduction in intracellular levels of ATP in PC12 cells. (A) Rotenone concentration-dependently decreased the intracellular levels of ATP in PC12 cells, assessed by a luciferin/luciferase-based ATP assay. PC12 cells were treated with 0.5 mM rotenone for 24 h. (B) NMN treatment attenuated the rotenone-induced reduction in the intracellular levels of ATP in PC12 cells. PC12 cells were treated with 0.5 mM rotenone, with or without co-treatment with NMN for 24 h. Subsequently, the intracellular levels of ATP in the cells were assessed by a luciferin/luciferase-based ATP assay. ^***^P<0.001 as indicated or vs. 0 mM rotenone; Con, control.

To investigate the mechanisms underlying the protective effect of NMN on rotenone-induced cell death, the effect of NMN treatment on the intracellular levels of ATP in rotenone-treated cells was determined. Rotenone concentration-dependently reduced the intracellular levels of ATP in the cells (Fig. 7A); this effect was significantly attenuated when the cells were co-treated with NMN (Fig. 7B).

## Discussion

The major results of the present study include: i) NMN attenuated the rotenone-induced reduction in the survival rate of PC12 cells; ii) NMN reduced the early- and late-stage apoptosis of rotenone-treated PC12 cells; iii) NMN restored the intracellular levels of NAD^+^ in rotenone-treated PC12 cells; and iv) the protective effects of NMN against rotenone-induced cell death were demonstrated through the prevention of rotenone-induced ATP depletion.

The results of the present study suggest that NMN attenuates cell apoptosis and decreases the intracellular levels of ATP in rotenone-treated PC12 cells. Cumulative evidence has suggested that NAD^+^ plays significant roles in a variety of biological processes, including energy metabolism, mitochondrial functions, calcium homeostasis, antioxidation/generation of oxidative stress, gene expression, immunological functions, aging and cell death (2). NAD^+^ treatment has also been found to decrease the rate of apoptosis of primary cultures of neurons, astrocytes and myocytes, induced by various insults (4). NAD^+^ acts as a neuroprotective agent via several mechanisms, including the prevention of mitochondrial impairment (4,21), prevention of ATP depletion and glycolytic inhibition (4,5,21), and the enhancement of DNA repair (6).

NMN is a major precursor of NAD^+^ in the salvage pathway of NAD^+^ synthesis, where it is converted to NAD^+^ in cells by nicotinamide mononucleotide adenylyltransferase (2). The current study demonstrated that NMN treatment was highly protective against the rotenone-induced cytotoxicity of PC12 cells in a cellular model of PD. This was revealed through various cell apoptosis assays, including LDH and MTT assays, and flow cytometry-based Annexin V/7-AAD and Hoechst staining. Furthermore, the present study demonstrated that NMN treatment was able to significantly decrease the rotenone-induced apoptosis of cells, as indicated by experiments that applied flow cytometry-based Annexin V and Hoechst staining, and Western blot analysis of PARP-1.

The mechanisms underlying the protective effect of NMN on the neurotoxicity of rotenone were investigated. The results of the present study demonstrated that treatment with NMN restored the intracellular levels of NAD^+^ and attenuated the reduction in the levels of ATP in rotenone-treated PC12 cells. Since intracellular ATP (22) and NAD^+^ (2) are mediators of cell survival, the beneficial effects of NMN on the levels of ATP and NAD^+^ may at least partially account for the protective effects of NMN against rotenone-induced cell death. As NAD^+^ restoration may lead to a reduction in the ATP consumption used for NAD^+^ synthesis (2), the NMN-induced restoration of intracellular levels of NAD^+^ may account for the beneficial effects of NMN on the intracellular levels of ATP.

Apoptotic changes are major pathological transformations in PD and numerous other neurological diseases (23,24). A compromise in energy metabolism may also play a significant role in the pathology of neurodegenerative disorders (9). The present study indicates that NMN treatment may be highly protective against not only apoptosis, but also energy compromise, in rotenone-treated PC12 cells.

These results suggest that NMN may become a promising drug for the treatment of PD and multiple other diseases in which apoptosis and energy compromise play significant pathological roles. Further *in vivo* studies of the effect of NMN on PD are required.
